# Role of IL-33 and Its Receptor in T Cell-Mediated Autoimmune Diseases

**DOI:** 10.1155/2014/587376

**Published:** 2014-06-16

**Authors:** Qing Zhao, Guangjie Chen

**Affiliations:** Department of Immunology and Microbiology, Shanghai JiaoTong University School of Medicine, Shanghai Institute of Immunology, 280 South Chongqing Road, Shanghai 200025, China

## Abstract

Interleukin-33 (IL-33) is a new cytokine of interleukin-1 family, whose specific receptor is ST2. IL-33 exerts its functions via its target cells and plays different roles in diseases. ST2 deletion and exclusion of IL-33/ST2 axis are accompanied by enhanced susceptibility to dominantly T cell-mediated organ-specific autoimmune diseases. It has been reported that IL-33/ST2 pathway plays a key role in host defense and immune regulation in inflammatory and infectious diseases. This review focuses on new findings in the roles of IL-33 and ST2 in several kinds of T cell-mediated autoimmune diseases.

## 1. Introduction

Interleukin-33 (IL-33) used to be named as “DVS27” [1]. In 2005, it was first reported that the gene sequence and structure of IL-33 were similar to those of IL-1*β* and IL-18 which belong to the IL-1 family [2]. So IL-33 is identified as a new member of the IL-1 superfamily [3]. It can activate mast cells, lymphocytes, and eosinophils to release the Th2-related cytokines [4, 5].

Autoimmune diseases result from an abnormal immune response to self-antigens to cause tissue damage. Patients with autoimmune diseases frequently have auto-reactive T cells and unusual antibodies circulating in their blood that target their own body tissues [6]. Common autoimmune diseases include rheumatoid arthritis (RA), systemic lupus erythematosus (SLE), ankylosing spondylitis, scleroderma, and Sjogren's syndrome. Currently, the exact pathogenesis of autoimmune diseases is still unclear [7]. It is thought that autoimmune disease is caused by different factors including genetic predisposition, environmental factors, and viral infection, which lead to abnormal immune response and loss of immune tolerance in the body.

It has been proven that IL-33 and its receptor play an important role in inflammation, infection, and autoimmune diseases. More recently, IL-33 has been shown to change the symptoms of rheumatoid arthritis, systemic lupus erythematosus, and other autoimmune diseases, which may elicit beneficial or detrimental effects depending on the disease setting [8, 9]. Therefore, studies on IL-33 may provide a new idea and target for the treatment of autoimmune diseases.

## 2. IL-33 & ST2

### 2.1. The Structure of IL-33

In 2003, Baekkevold et al. firstly identified IL-33 from the high endothelial cells; its protein sequence was similar to DVS27 of canine [10]. DVS27 gene was found to encode a nuclear protein that could be involved in inflammatory events. Then in 2005, Schmitz et al. found that IL-33 was related to IL-1 and fibroblast growth factor by using of a computational approach to search sequence databases for proteins [2]. The human IL-33 gene is located on chromosome 9p24.1 and encodes a peptide of 270 amino acids, while this gene maps to chromosome 19qC1 and encodes a peptide of 266 amino acids in mice. IL33 is a 30 kD protein that is evolutionarily conserved in mammals, with 54% amino acid sequence identity between the human and mouse homologs [11]. IL-33 is a member of the IL-1 superfamily of cytokines, a determination based in part on the molecules *β*-trefoil structure, a conserved structure type at the carboxyl terminal [12], through which IL-33 exerts its cytokine activity [13]. IL-33 was initially believed to be cleaved by caspase-1 to release a “mature” 18 kD form corresponding to the C-terminal cytokine domain. However, it soon became clear that the IL-33 protein only contains the cleavage sites of caspase-3 and caspase-7 but not the cleavage sites of caspase-1 [14, 15]. Recent studies showed that neutrophil-derived caspase and elastase also cleave IL-33 but generate more bioactive cytokines, such as IL-1*α*, IL-1*β*, and IL-18 [16]. The findings highlight the major differences between the biology of IL-33 and that of IL-1*β* [17, 18].

### 2.2. IL-33 Receptor

IL-33R is a heterodimer comprised of IL-1RL1 (also called ST2) and IL-1 receptor accessory protein (IL-1RAcP) [19]. Regarding ST2, its gene was found in 3T3 cell lines derived from BALB/c mice in 1989. The research showed that ST2 was expressed on growth-stimulated BALB/c-3T3 cells but not in resting cells [20]. ST2 gene is located on human chromosome 2 (2q12), its germline sequence is conserved [21]. ST2 is a member of the IL-1 family, which has the Toll/IL-1R domain in the cytoplasmic region. ST2 has 2 major forms: sST2 and ST2L [22]. sST2 is a soluble ST2 which has no transmembrane sequence, so it can be excreted outside the cells, while ST2L is the transmembrane ST2 for having transmembrane sequence; both of them are produced from the IL-1RL1 gene as a result of alternative splicing under the control of two distinct promoters [23, 24]. The transmembrane-form ST2L is considered to be a functional component of IL-33R, whereas sST2 is regarded as a decoy receptor for IL-33, like soluble IL-1R for IL-1*α* and IL-1*β* [25]. Compared with sST2, the structure of ST2L is more similar to IL-1R. What is more, ST2V and ST2LV are two splice variants of ST2. Loss of the third immunoglobulin motif and alternatives splicing in the C-terminal portion of ST2, resulting in a unique hydrophobic tail, produces ST2V, whereas alternative splicing, leading to deletion of the transmembrane domain of ST2L, produces ST2LV [26].

### 2.3. Physiological Functions

IL33 is a dual-function protein that acts as both a cytokine and a nuclear factor. In some mice study, it is constitutively expressed by the epithelial and endothelial cells of many organs [27], and it is also expressed by some innate immune cells such as dendritic cells and macrophages [28, 29]. IL-33 can increase the expression of IL-5 and IL-13 in Th2 cells [30, 31], but IL-33 cannot improve the expression level of IL-4 [32]. It implies that IL-33 is not necessary for the differentiation of Th2 cells. Native CD4 and Th1 cells do not express IL-33R, culture of resting Th2 cells in medium containing IL-33 combined with IL-2, IL-7, or TSLP, and cause upregulation of IL-33R expression [32].

IL-33 is a ligand that binds to a high affinity receptor family member ST2 [33]. ST2 expresses on the surface of both mouse and human mast cells [34]. Without causing degranulation, IL-33 can induce mast cells to produce chemokines and cytokines. But some other studies showed that IL-33 could promote IgE mediated cytokine production and cause degranulation of mast cells in mice [35]. IL-33 can also induce basophils to produce Th2-type cytokines and chemokines at the same time and then increases the expression of cell adhesion molecules and CD11b on basophils both in human and mice [36, 37].

It was reported that Th2 lymphocytes, mast cells, macrophages, dendritic cells, CD8 T cells, and B cells and some granulocytes such as basophils and eosinophils are possible to express membrane-type ST2 molecules [34, 38, 39]. IL-33 could induce the production of many cytokines such as IL-5, IL-13, TNF-*α*, IFN-*γ*, and IL-2 when binding with ST2 receptors on the surface of those cells [40, 41]. Therefore, IL-33 and ST2 play an important role in allergic diseases and various mucosal immune responses, suggesting the involvement of the IL-33/ST2 pathway in the pathogenesis of a large number of diseases, especially in the responses based on the Th2 type [5, 37].

Recently, group 2 ILCs, which include IL-5- and IL-13-producing ILCs such as natural helper cells (NHCs), nuocytes, and innate helper type 2 (Ih2) cells, are reported to be critically dependent on the epithelial cell-derived cytokines IL-33 and IL-25 and have been shown to promote anti-helminth immunity and regulate inflammation and/or epithelial repair in the lung [42]. And it is also found that IL-33, which is released from necrotic cells, is necessary for potent CD8+ T cell (CTL) responses to replicating prototypic RNA and DNA viruses in mice [39].

Accumulating evidence suggests a crucial role of IL-33/ST2 in inducing and modifying host immune responses against a variety of pathogens including parasites, bacteria, viruses, and fungi as well as sterile insults of both endogenous and exogenous source [43].

DCs are the major antigen-presenting cells and play a pivotal role in immune responses. DCs respond directly to IL-33 through ST2 [44]. DCs not only respond to IL-33 but also produce IL-33 in allergic condition [45]. The interaction between IL-33 and DCs may represent a new pathway to initiate Th2-type immune responses.

In addition, ST2 blocking antibody or IgG2-ST2 fusion proteins can activate Th1 cells to enhance Th1 cell responses [46], while they inhibit the allergic airway inflammation caused by the Th2 cells [47].

### 2.4. IL-33/ST2 Signaling Pathway

Kakkar's study has shown that IL-33/ST2 signaling pathway was involved in T cell-mediated immune response and was a potential medium for a variety of inflammatory diseases. IL-33 may function as a modulator of NF-*κ*B and canonical Toll-like receptor/IL-1 receptor signaling [48].

**Figure 1:**
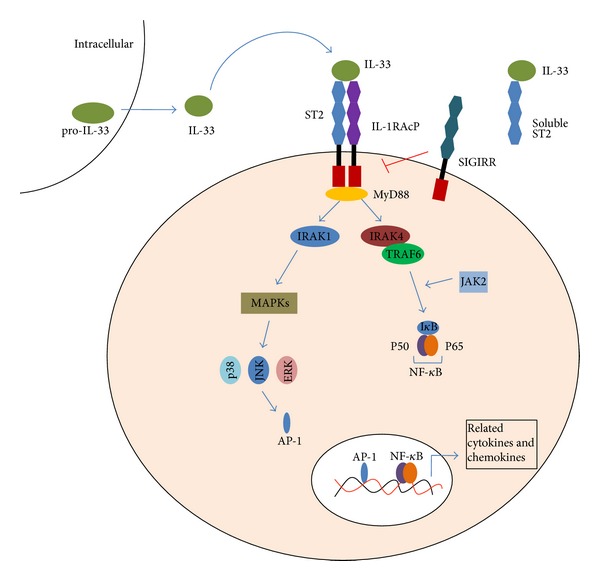
IL-33/ST2 signaling pathway. IL-33 is the ligand for ST2. It activates the ST2L/IL-1RAcP dimers or is neutralized by binding to sST2. The interaction of IL-33 with ST2 leads to the recruitment of the myeloid differentiation primary-response protein 88 (MyD88), IL-1R-associated kinase 1 (IRAK1) and IRAK4, which result in the activation of at least two independent pathways: the transcription factor nuclear factor-*κ*B (NF-*κ*B) and the mitogen-activated protein kinase (MAPK) pathway, ultimately induce related gene expression. It induces the production of IL-2, IL-5, IL-13, TNF-*α*, and IFN-*γ*. IL-33 can also combine with single Ig IL-1R-related molecule (SIGIRR), which seems as an inhibitor of IL-33/ST2 pathway.

As shown in Figure 1, IL-33 can transfer extracellular information by binding with the ST2 receptor complex when serving as a conventional cytokine [43]. Furthermore, IL-33 can act as an intracellular nuclear factor which inhibits the transcription. Nuclear IL-33 is thought to be involved in transcriptional repression by binding to the H2A-H2B acidic pocket of nucleosomes and regulating chromatin compaction by promoting nucleosome-nucleosome interactions. However, the specific transcriptional targets or the biological effects of nuclear IL-33 are unclear at present [49]. Based on the previous researches, it has been recognized that IL-33 signaling pathways are different from the typical Th2 cytokines mediated pathway.

IL-33 binds with the receptor complex containing ST2 and IL-1RAcp and acts through the Toll/IL-1 receptor domain of IL-1 receptor accessory protein (IL-1RAcp), which is shared by other IL-1 family members such as IL-1R and IL-18R, to transduce the IL-33/ST2 signal [50, 51]. This causes the recruitment of MyD88 (myeloid differentiation primary response gene 88), IRAK (interleukin-1 receptor-associated kinase 1), and IRAK4 (interleukin-1 receptor-associated kinase 4) by the intracellular TIR domain of IL-1RAcP [52]. Then the complex activates the transcription factor NF-*κ*B and AP-1 through mitogen activated protein kinases (MAPK) or TNF receptor associated factor 6 (TRAF6), finally causing inflammatory responses [2].

Single Immunoglobulin IL-1 Receptor-Related molecule (SIGIRR) which is also named as the Toll-Interleukin-1 Receptor 8(TIR8) belongs to the IL-1 receptor family. It has been reported to be a negative regulator in IL-1R and Toll-like receptor-mediated immune responses [72]. SIGIRR could be formed into a complex together with the ST2 on the membrane. Then the new combined complex could inhibit IL-33/ST2 signaling pathway [73, 74] (Figure 1). Some studies showed that the dendritic cells of SIGIRR gene deficient mice performed higher immunoreactivity after stimulation with IL-1, IL-18, and TLR agonists [75]. In addition, the level of Th2 cytokines was increased in Th2 cells after IL-33 stimulation in SIGIRR-deficient mice [74].

## 3. IL-33/ST2 Axis in Autoimmune Diseases 

IL-33 exerts its functions via its target cells and plays different roles in diseases. IL-33 has been implicated in a wide range of immune-mediated diseases, including allergic, cardiovascular, and autoimmune conditions, which, depending on the disease setting, may elicit beneficial or detrimental effects [43]. ST2 deletion and exclusion of IL-33/ST2 axis are accompanied by enhanced susceptibility to dominantly T cell-mediated organ-specific autoimmune diseases [76].  Table 1 lists the major findings in the context of IL-33 and ST2 in autoimmune diseases.

### 3.1. IL-33/ST2 Axis in Rheumatoid Arthritis

Rheumatoid arthritis (RA) is a chronic systemic inflammatory disease characterized by the immune cell mediated destruction of the joint architecture [77]. Elevated levels of proinflammatory cytokines are a key feature in patients with RA.

The majority of IL-1 family members currently known are proinflammatory cytokines in RA [78]. It has been shown that IL-33 was highly expressed in the synovium of RA patients by in situ hybridization experiments [53]. And IL-33 expression was increased in inflammation parts of collagen-induced arthritis model in mice. Meanwhile, local high expression of IL-33 is the pathological basis of joint inflammation and bone destruction [79]. The observations suggested that inhibition of IL-33R may have the same role in the treatment of rheumatoid arthritis which is similar to IL-1 suppressor pathway [80]. What's more, treatment of anti-TNF-*α* antibody (infliximab) dramatically decreased the level of IL-33 in clinical therapy of RA patients, which is more effective than treatment with methotrexate alone. Therefore, the measurement of IL-33 production in serum could be a distinct marker for monitoring the efficacy of RA treatment.

In the synovium of RA patients, IL-33 was mainly expressed in vascular endothelial cells, fibroblasts, and some CD68+ mononuclear inflammatory cells [46, 81], while the synovial fibroblasts had little or even no expression of IL-33 [82]. In addition, many studies showed that IL-33 levels were significantly increased in synovial fluid and serum of RA patients compared with healthy controls, suggesting that high levels of IL-33 may be associated with the pathogenesis of RA [54, 83]. Another study pointed out that there is no correlation between the IL-33 concentration and disease activity of RA, but it was positively correlated with autoantibody levels, suggesting that IL-33 may be related with autoantibodies in RA [53].

Xu's group found that with the administration of IL-33 in collagen-induced arthritis, the aggravated symptoms of RA appeared in wild type mice, and proinflammatory cytokines and anticollagen antibodies were elevated too. In contrast, these phenomena were not found in ST2 genetic deficient mice. Notably, when transferring mast cells to the ST2 genetic deficient mice, the above situation was reversed, both the symptoms and expression of proinflammatory cytokines were the same as those of the wild type. This implied that IL-33 exacerbates antigen-induced arthritis by acting on mast cells [82]. Verri et al. found the aggregation of neutrophil in the arthritis model of mice during which accompany the increased expression of IL-33 mRNA and IL-33R, whereas IL-33R deficient mice were less susceptible to arthritis invasion. More reports demonstrated that inhibiting the IL-33R expression of neutrophil could prevent the migration of neutrophil induced by IL-33, and which might be the important mechanism of the effect of anti-TNF-*α* on RA. Administration of sST2 which could neutralize IL-33 on collagen-induced arthritis in rats can reduce arthritis significantly, including reducing the clinical symptoms, synovial hyperplasia, and joint erosion. The mechanism is that sST2 can reduce the level of IL-6, IL-12, and TNF-*α* by neutralizing IL-33, thereby ameliorating the pathological symptoms in antigen-induced arthritis of rats in vivo [84]. In anti-ST2-treated mice, draining lymph node cells produced less IL-17 compared with the control group indicating that IL-33 is able to enhance Th17 responses. Recently, a new study [55] reported that endogenous IL-33 was not necessary for the development of joint inflammation in autoantibody-induced arthritis of IL-33-deficient mice. But it was also found that ST2-deficient mice had reduced arthritis severity in that study. This research indicates an IL-33-independent effect of ST2.

Taken together, these data imply that the IL-33/ST2 pathway plays a critical role in the pathogenesis of rheumatoid arthritis.

### 3.2. IL-33/ST2 Axis in Multiple Sclerosis

Brain and spinal cord are the organs which have the highest expression of IL-33 [2]. Astrocytes are the nonhematopoietic epithelial cells in the central nervous system (CNS), and they could express IL-33 receptor 2 subunit ST2 and IL-1RAcP [85, 86]. Therefore, CNS glial cells, particularly astrocytes, can possibly be activated by IL-33. IL-33 can induce proliferation of microglia and also improve the expression of proinflammatory cytokines IL-1*β* and TNF-*α*, while increasing the expression of anti-inflammatory factor IL-10 at the same time. Microglial cells secrete various cytokines and chemokines, which are a major regulator of the central nervous system [87].

Multiple sclerosis is an inflammatory disease of the CNS, characterized by inflammatory lesions, demyelination, and axonal loss. Based on the findings between IL-33 and CNS cells, many studies have shown that IL-33 played an important role in MS [88]. MS lesions are restricted to the central nervous system and are characterized by infiltration of lymphocytes, macrophages, and dendritic cells [89]. These cells can cause inflammatory cytokine gene expression and the formation of toxic protein to promote damage in CNS. IL-33/ST2 signal pathway could activate the gene of NF-*κ*B, STAT1, and STAT6, thereby contributing to demyelination [56, 57]. In addition, the mast cells of MS patients are an important kind of inflammatory cells. IL-33 can activate the mast cells in CNS by degranulation and inducing the production of a number of proinflammatory cytokines [4]. Christophi et al. found that, compared with normal subjects, the expression mRNA and protein level of IL-33 are remarkably higher in plasma, CNS lesions, and normal white matter (NAWM) of MS patients [58]. It was also found that NF-*κ*B expression level was significantly increased in the peripheral blood mononuclear cells and macrophages in vitro from MS patients [58]. Furthermore, treatment of IFN-*β* can simultaneously reduce the expression of NF-*κ*B and IL-33 both in vivo and in vitro [90]. The viral or bacterial components such as lipopolysaccharide and flagellin owe the ability to induce NF-*κ*B expression to the Toll-like receptor3 and Toll-like receptor5 [91], and viral infections can induce astrocytes secreting IL-33 [92]. Therefore, IL-33 plays the regulatory role by feedback mechanism in the pathogenesis of MS [59].

Experimental autoimmune encephalomyelitis (EAE) is an inflammatory demyelinating disease of the central nervous system that serves as a classic animal model for MS. Recent studies have found that the level of IL-33 is higher in the CNS of EAE mice compared with the normal controls [60]. The significantly raised level of IL-33 from endothelial cells and astrocytes exert vital regulating action in CNS. By increasing of IL-33, the activity of astrocytes was affected subsequently, ultimately resulting in the proliferation of microglia and secretion of cytokines and chemokines [87]. During the inductive phase of EAE, IL-33 administration aggravates the disease, while anti-IL-33 antibody significantly inhibits the onset and severity of EAE by reducing MOG35-55 specific stimulated production of IFN-*γ* and IL-17 [61].

However, some completely opposite findings have been reported. Jiang's group demonstrated that IL-33 administration after EAE onset is protective against the disease. The effect of IL-33 is likely endogenous as ST2^−/−^ mice developed exacerbated disease following EAE induction. Furthermore, the beneficial effect of IL-33 is accompanied by a switch from the predominant pathogenic Th17 and Th1 response to the protective Th2-type immune activity. Moreover, IL-33 treatment also resulted in an increase in regulatory T cell (Treg) frequency and the polarization of alternative macrophages, which has been previously demonstrated to be associated with a favorable outcome of EAE [62]. Another study also showed that BALB/c mice developed highly inflammatory T helper cells by targeting ST2 gene. These cells were able to enter CNS, produced inflammatory cytokines, and disturbed blood brain barrier, then enabled influx of other immune cells in CNS and caused EAE in the absence of ST2 molecule in contrast to WT mice [63].

Although the reason for the difference in the above findings is still not known and the exact pathophysiological function of the IL-33/ST2 axis in MS requires further investigation, it's believed that IL-33/ST2 axis plays some important role in MS development. It is possible that IL-33 may become a potential biomarker for MS in the future, as well as the target of MS treatment.

### 3.3. IL-33/ST2 Axis in Systemic Lupus Erythematosus

Systemic lupus erythematosus (SLE) is a multisystemic autoimmune disease, characterized by hyper gamma globulinemia and a plethora of autoantibodies as well as proinflammatory cytokines. Both Th1 and Th2 responses have been implicated in the pathogenesis of lupus [64].

The studies have pointed out that the level of IL-33 is abnormal in the serum of SLE patients, suggesting that IL-33 may be involved in the pathogenesis of SLE [65]. The studies also analyzed the relationship between IL-33 and the kidney damage in SLE as well as disease activity.

Mok's group demonstrated the sST2 level was higher in SLE patients with active disease including renal or nonrenal manifestations compared with those with lesser disease activity and normal controls. In view of the significant correlation between serum sST2 level and parameters of disease activity, its responsiveness to change in the levels of disease activity when monitored longitudinally and its lack of association with age, sex, and impaired renal function, sST2 may serve as a potential surrogate marker for disease activity in SLE [66]. In Yang's study, it was found that the level of IL-33 in serum, which was not influenced by gender or age, were significantly increased in patients with SLE, compared with healthy people [93].

In patients with SLE, most clinical and laboratory characteristics did not correlate with serum IL-33 levels, with exceptions of thrombocytopenia, erythrocytopenia, anti-SSB antibody, ESR, CRP, and IgA. Patients with SLE showed close correlation of IL-33 with ESR, CRP, and IgA [93]. Moreover, IL-33 may exert biologic effects on erythrocytes and platelets or their precursors in SLE. As it is known to all, ESR and CRP are diagnostic indicators which represent the SLE patients being in the acute inflammation phase. The results suggest that IL-33 may be involved in the acute inflammation phase of SLE, but it was not associated with course of the disease.

Sigrr acts as a novel SLE susceptibility gene in mice; lack of Sigrr enhanced the activation and proliferation of B cells, including the production of autoantibodies against multiple nuclear lupus autoantigens [94].

### 3.4. IL-33/ST2 Axis in Inflammatory Bowel Disease

Inflammatory bowel disease (IBD) is chronic recurrent disease, including ulcerative colitis (UC) and Crohn's disease (CD). The etiology and pathogenesis are not clear, but it is known that inflammatory response caused by abnormal intestinal mucosal immune system plays an important role in the pathogenesis of IBD.

In recent years, scientific interest in the significance of the IL-33/ST2 system in IBD physiopathology has grown [95]. In 2010, five different research groups consistently described the dysregulation of IL-33 in patients with inflammatory bowel disease (IBD) [67–70, 96]. And the IL-33 level correlates with UC activity. The results showed that a specific increase of mucosal IL-33 in active UC localized primarily to intestinal epithelial cells (IEC) and colonic inflammatory infiltrates. Infliximab (anti-TNF) treatment of UC decreased circulating IL-33 and increased sST2 level, whereas stimulation of HT-29 IEC by TNF in vitro results in increase of IL-33 and sST2 [69]. IL-33 was increased and correlated with disease severity, further induced IL-5, IL-6, and IL-17 production from mesenteric lymph node (MLN) cells, and displayed a similar pattern of mucosal cell production as IBD patients [71].

Animal models of IBD were subsequently utilized in other studies in order to mechanistically determine the precise role of IL-33 in chronic intestinal inflammation. It is found that IL-33/ST2 pathway might possess dichotomous functions. One is to enhance inflammatory responses, the other is to promote epithelial integrity. In two experimental models of IBD, genetic ablation of ST2 significantly improved signs of colitis, while a sustained epithelial expression of the cyto-protective factor connexin-43 was observed in DSS-treated ST2-deficient mice. Absence of ST2-mediated signaling in nonhematopoietic cells protects against Dextran sulfate sodium (DSS) induced colitis. In addition, IL-33 treatment impaired epithelial barrier permeability in vitro and in vivo, whereas absence of ST2 enhanced wound healing response upon acute mechanical injury in the colon [97]. It is also reported that IL-33 reduced disease severity by modulating Th1 inflammation and induced a shift to Th2-associated cytokine production in this model [98].

According to the results of a new survey, IL-33 and ST2 genes are early induced in the colonic tissue during DSS-induced colitis. Furthermore, IL-33 exacerbates acute colitis in association with the induction of proinflammatory and angiogenic cytokine as well as chemokine production in a ST2 and IL-4 dependent manner [99].

Recent data which were obtained in an Italian cohort of adult and pediatric UC and CD patients demonstrated that specific IL-33 and ST2 gene polymorphisms confer an increased risk of developing IBD (both UC and CD), suggesting the involvement of the IL-33/ST2 axis in the onset of chronic intestinal inflammation [100]. Garlanda et al. reported that TIR8 represents a negative pathway regulation of the IL-1 receptor/TLR system, expressed in epithelial cells and DC, crucial for tuning inflammation in the gastrointestinal tract [75].

All these findings may lead to exciting potential therapeutic strategies for IBD patients.

## 4. Conclusion

As a kind of proinflammatory cytokines, IL-33 cause the immune pathological damage in some tissues [101]. It plays the important role in chronic inflammatory and autoimmune diseases. It has been proven that IL-33/ST2 signal transduction pathways are involved in various pathological processes. IL-33 and its receptor ST2 are the potential targets for the treatment of allergic and autoimmune inflammatory diseases. However, their exact roles in these diseases still remained poorly understood.

Therefore, the needs for more researches in molecular biology, genetics, immunology, and other related disciplines are urgent. And further study of IL-33 and ST2 structure, function, and signal transduction pathways involved in autoimmune diseases is required. These will provide new ideas for the treatment of autoimmune diseases and other diseases.

## Figures and Tables

**Table 1:** Dysregulated expression of IL-33 and ST2 in some autoimmune diseases.

Disease	Major findings	Reference
RA	Levels of IL-33 and ST2 are increased in serum, synovial fluid, and synovium.	[53–55]
	Level of serum IL-33 correlates with RA-related autoantibodies but not RA severity.	
	IL-33 enhances Th17 response.	

MS	IL-33/ST2 signal pathway activates NF-*κ*B, STAT1, and STAT6, thereby contributing to demyelination.	[56–63]
	Level of IL-33 is increased in the spinal cord of MS patients.	
	Levels of IL-33 and ST2 are increased in the spinal cord of EAE.	
	Contradictory functions of IL-33 in EAE: IL-33 attenuates EAE but IL-33 administration aggravates EAE.	

SLE	Levels of IL-33 and ST2 are increased in serum in SLE patients.	[64–66]
	IL-33 correlates with ESR and CRP, but not the kidney damage disease activity in SLE patients.	
	Serum ST2 correlates with severity of SLE.	

IBD	Levels of IL-33 and ST2 are increased in serum.	[67–71]
	Level of IL-33 is increased in mucosa.	
	IL-33 correlates with UC activity and IBD severity.	
	IL-33 induces IL-5, IL-6, and IL-17 production.	

## List of Abbreviations

CD: Crohn's disease
CNS: Central Nervous System
CRP: C-Reactive Protein
EAE: Experimental Autoimmune Encephalomyelitis
ESR: Erythrocyte Sedimentation Rate
IBD: Inflammatory Bowel Disease
IEC: Intestinal Epithelial Cells
IL-1RAcP: IL-1 Receptor Accessory Protein
IRAK: Interleukin-1 Receptor-Associated Kinase
MAPK: Mitogen Activated Protein Kinases
MLN: Mesenteric Lymph Node
MS: Multiple Sclerosis
MyD88: Myeloid Differentiation Primary Response Gene 88
RA: Rheumatoid Arthritis
SIGIRR: Single Immunoglobulin IL-1 Receptor-Related Molecule
SLE: Systemic Lupus Erythematosus
TRAF6: TNF Receptor Associated Factor
UC: Ulcerative Colitis.
